# Oatmeal Diet for Diabetics

**Published:** 1909-11-06

**Authors:** 


### MEDICINE.

### OATMEAL DIET FOR DIABETICS.

It is to Von Noorden that we owe the suggestion that it is possible to give certain diabetic patients food prepared from oatmeal, even when they are unable to take other forms of carbohydrate food at all, though of course there are some patients to whom even oatmeal is harmful. The latter can only be determined by trial in each individual case, since the severity of the symptoms themselves and the amount of glycosuria afford little or no indication as to whether oatmeal will be well tolerated or no.

The good results in certain cases are remarkable. As an instance in point we may quote Dr. Herrick's patient, a man of 37 years of age, who had lumbar pains and a small ischio-rectal abscess, and who, though he had not lost weight or noticed polyuria, had for several years past been thirsty enough to drink more water than other people, and had also been subject to boils. The urine passed in every 24 hours amounted to over a litre and a half, and it contained 108 grams of sugar.

Ordinary diabetic diet to which wheaten carbohydrate, to the extent of two slices of bread was added, only brought the sugar down to 98 grams per diem. When oatmeal was added to the extent advocated by Von Noorden the sugar in ten days' time fell to 1½ gram per diem.

It seems that even in the severe forms of diabetes that occur in young subjects good results are to be obtained in this way. In these cases a strict diabetic dietary may bring about much improvement, but the greatest relief to the thirst, the polyuria, and the glycosuria may result from the adoption of the oatmeal diet. Von Noorden advocated 250 grams of oatmeal, from 250 to 300 grams of butter, and 100 grams of proteid as a basis for his dietary. The oatmeal may be cooked in many different ways, one of which is to simmer it in water for about two hours, butter and the white of eggs being well stirred in with the oatmeal when it is nearly cooked, salt also being added to taste.

It might be thought that this diet would be too monotonous to be maintained for any length of time. This apparently is not the case in practice, and in selected patients, not only has the power of tolerating carbohydrate been so much augmented that the patient can take an amount of starch which would previously have made the general condition worse, but also the danger of impending coma is actually diminished by the oatmeal diet because it leads to a diminution in the toxic acetone bodies that are present in the blood and in the urine in these cases.

